# Low-intensity online mindfulness-based intervention for university students with anxiety during the COVID-19 pandemic—A randomized controlled trial with 3-month follow-up

**DOI:** 10.1016/j.invent.2023.100665

**Published:** 2023-09-11

**Authors:** Daniel Kim-wan Young, Per Carlbring, Petrus Yat-nam Ng, Daphne Yi Ting Cheng, Joseph Qi-rong Chen, Siu-man Ng

**Affiliations:** a Department of Social Work, City University of Hong Kong, Hong Kong; b Department of Psychology, Stockholm University, Stockholm, Sweden; c Department of Social Work, Hong Kong Baptist University, Hong Kong; d Department of Social Work & Social Administration, University of Hong Kong, Hong Kong

**Keywords:** Anxiety, Chinese, Online mindfulness-based intervention, Randomized controlled trial, University students

## Abstract

**Objective:**

This study investigated the effectiveness of a low-intensity online mindfulness-based Intervention (iMBI) for alleviating anxiety in university students during the COVID-19 pandemic.

**Methods:**

In a randomized controlled trial involving 134 participants from a local university in Hong Kong, subjects were randomly assigned to either the intervention group (*n* = 67) or the inactive control group (*n* = 67). The intervention group participated in a low-intensity iMBI comprising 16 online modules and two half-day online mindfulness workshops over an eight-week period. Outcomes were measured via an online platform using standardized assessment scales, including the Beck Anxiety Inventory and the Chinese Five Facets Mindfulness Questionnaire, at three different time points: pre-intervention, post-intervention and at a three-month follow-up.

**Results:**

Intent-to-treat analysis using 2 (group) × 3 (time) repeated measures of covariance (ANCOVA) showed that the intervention group, compared to the control group, showed a significant reduction in anxiety symptoms with a medium effect size (Cohen's *d* = 0.5) and a significant improvement in mindfulness skills with a medium effect size (Cohen's *d* = 0.5) at post-intervention. The effects of the intervention in reducing anxiety and improving mindfulness persisted at the three-month follow-up.

**Conclusion:**

The results of this study demonstrate the effectiveness of the low-intensity iMBI in alleviating anxiety among university students.

## Highlights

• The online mindfulness-based intervention yielded a reduction in anxiety with a medium effect size; and an improvement in mindfulness skills with a small to medium effect size at post-intervention.
• The treatment effects, in terms of reducing anxiety and enhancing mindfulness, were sustained at the three-month follow-up

## 1 Introduction

Mental disorders, including anxiety, are highly prevalent among university students globally, both prior to the COVID-19 pandemic (Auerbach et al., 2016) and during it (Liyanage et al., 2022). In Hong Kong, university students have reported higher levels of stress compared to their counterparts in other societies (Lun et al., 2018). During the COVID-19 pandemic, the prevalence rate of anxiety among these students reached 50.00 % (Hung et al., 2022; Shek et al., 2022). This rate was notably higher than that of the general population in Hong Kong (14 %; Hou et al., 2021), and university students in most countries (40 %; Liyanage et al., 2022).

Despite this, many university students are reluctant to seek traditional face-to-face counselling due to a number of barriers. These include local social distancing, limited accessibility to traditional counselling services and associated stigma (Young et al., 2022b). To circumvent these issues, the Internet-delivered Mindfulness-based Intervention (iMBI) has been developed to allow individuals to learn mindfulness techniques to manage their anxiety at home via the internet and mobile apps (Witarto et al., 2022). Research reviews have shown that iMBIs can be effective in reducing anxiety both the pre-pandemic (Sevilla-Llewellyn-Jones et al., 2018) and during the pandemic (Witarto et al., 2022). However, there has been a limited number of rigorous studies of iMBIs for university students during the pandemic (Witarto et al., 2022). Specifically, only three randomized controlled trials of iMBIs for university students have been conducted in Germany (Karing, 2022), the UK (Simonsson et al., 2021) and mainland China (Sun et al., 2022), respectively.

This highlights the need for more randomized controlled trials. Given the lack of standardization of iMBIs across countries, there are also considerable variations among iMBI programs in terms of delivery methods, number of sessions/modules, level of counsellor support and theoretical approaches (Sommers-Spijkerman et al., 2021). It is therefore crucial to develop an iMBI that is feasible, acceptable and effective in the local context.

During the COVID-19 pandemic in Hong Kong, a new low-intensity iMBI for university students was developed in May 2022 (see https://imbihk.com). Briefly, this low-intensity iMBI provided sixteen self-guided online modules via an online platform that were accessible anywhere, anytime, and anonymously, and two half-day online mindfulness workshops delivered via the Zoom Apps over an eight-week period (see below for more details).

This study aimed to assess the effectiveness of the above low-intensity iMBI for university students experiencing anxiety. It was hypothesized that the intervention group, after participating in the low-intensity iMBI, would exhibit a significantly greater reduction in anxiety symptoms and improvement in mindfulness compared to the non-active control group. The study also sought to investigate the intervention's effect on promoting clinical improvement. Furthermore, characteristics of participants who benefited most from the low-intensity iMBI were examined, as limited studies were done in this area.

## 2 Methods

### 2.1 Study design

The study employed a randomized controlled trial design. The low-intensity iMBI was actively advertised to undergraduate and master's students at a local university in Hong Kong via mass email. Eligible participants, screened using online assessment tools, were randomly assigned in a 1:1 ratio to either the intervention or non-active control group. This allocation was made by the first author, who did not conduct the pre-intervention assessment or the online mindfulness workshop. The intervention group participated in low-intensity iMBI over an eight-week intervention period. The non-active control group received no intervention due to limited staff resources during the pandemic. All participants completed identical online outcome assessment scales at three time points: pre- intervention (T0), post- intervention (T1) and three months after the completion of the intervention (T2). Participants were informed of their results at T0, T1 and T2. Those who had severe mental health problems received an automated email advising them to seek mental health services and relevant contact information. In addition, participants were referred to the university counselling centre and/or crisis intervention services if needed. Ethical considerations in this study were reviewed and approved by the Hong Kong Baptist University Research Committee [reference number: REC/19-20/0488]. Written informed consent was obtained from all participants during the initial online screening. Data were collected from June 2022 to April 2023.

### 2.2 Subject inclusion criteria

To be eligible for the study, participants needed to be (1) 18 years or older, (2) currently experiencing minimum to moderate anxiety as assessed by the Chinese Beck Anxiety Inventory (BAI score ≥ 5; Zhou et al., 2018), (3) a university student, and (4) have access to a computer and/or smartphone and capable of independently managing online modules. Participants were excluded if they had a psychotic disorder, substance use disorder, severe anxiety or depression, or were at risk of suicide.

### 2.3 Sample size

The sample size was estimated using G*Power 3.1 software (Faul et al., 2007). This study aimed to identify an effect size of Cohen's *d* = 0.35 with a power of 80 %, necessitating a minimum sample size of 80 subjects. Given the anticipation of a high dropout rate of 40 %, the study planned to recruit 134 subjects.

### 2.4 Intervention

This intervention consisted of sixteen self-guided online modules that were adapted from Mindfulness-based Cognitive Therapy (Segal et al., 2013). They aimed to enhance awareness of habitual negative thinking patterns, promote presence in the current moment rather than ruminating, foster acceptance of emotional distress, and encourage a sense of hope in the face of stressful situations (Segal et al., 2013). These modules were further tailored to address the specific needs of university students during the pandemic, fostering attitudes of “going with the flow of life”, “living with physical discomfort”, and “enduring emotional distress”. These modifications were intended to help participants cope with the unexpected life changes and challenges brought about by the pandemic. The self-guided online modules covered various topics: psychoeducation and normalization of anxiety experiences (Module 1); introduction to mindfulness and automatic pilot (Modules 2–3); learning and practicing basic mindfulness exercises such as mindful eating, mindful sketching, body scan, and mindful breathing (Modules 4–6); living with physical discomfort (Modules 7–8); coping with emotional distress (Modules 9–10); embracing imperfection (Modules 11–12); finding compassion, mercy, and blessings in life (Modules 13–15); and integrating mindfulness into everyday life, along with a summary (Module 16).

Using a newly developed online platform (see https://imbihk.com) that could be accessed anywhere, anytime and anonymously, the intervention group was asked to complete two modules per week at home, each module lasting about 30 min. Each module included texts and video introducing key concepts of a topic, videos demonstrating mindfulness skills, reflection questions and audio-visual materials to facilitate mindfulness practice.

In addition, each participant in the intervention group was encouraged to attend two half-day online workshops on mindfulness via the Zoom app during the second and sixth weeks of the intervention period. These workshops were led by an experienced social worker who had completed training in mindfulness-based cognitive therapy (Segal et al., 2013). In addition, this intervention provided weekly reminders and technical support to use the online modules via asynchronous text messages (e.g. WhatsApp) and email, with each contact lasting <10 min.

### 2.5 Outcome assessment tools

The primary intervention outcome was the reduction of anxiety, as measured by the 21-item Chinese Beck Anxiety Inventory (BAI; Zhou et al., 2018). Each item (e.g. difficulty in breathing) is rated on a 4-point Likert scale ranging from 0 (not at all) to 3 (severely). The scale has shown excellent validity and reliability (Cronbach's *α* = 0.94; Zhou et al., 2018). The score of BAI was summed across all items, with possible scores ranging from 0 to 63. 0–7 represents minimal/no anxiety, 8–15 represents mild anxiety, 16–25 represents moderate anxiety, and 26–63 represents severe anxiety. A cut-off point of ≥16 is indicative of clinical anxiety.

The secondary intervention outcome was improvement in mindfulness, as measured by the 39-item Chinese Five Facets Mindfulness Questionnaire (FFMQ; Hou et al., 2014). Each item (e.g. when I have distressing thoughts or images, I just notice them and let them go) is rated on a 5-point Likert scale ranging from 1 (never) to 5 (very often). The scale has shown adequate internal consistency (Cronbach's *α* = 0.80) and test–retest reliability (*r* = 0.88) (Hou et al., 2014). The score of the FFMQ was summed across all items, with possible scores ranging from 0 to 195. A higher score indicates a higher level of mindfulness.

### 2.6 Data analyses

Two-tailed *p*-values of <0.05 were considered statistically significant. Statistical analyses were performed using SPSS 29.0 (IBM Corporation, 2022). Within-group intervention effects for both the intervention and control groups were investigated by using paired-sample *t*-tests on outcome assessment scores, i.e. BAI and FFMQ. Between-group intervention effects on outcome assessment scores were investigated using repeated measures analysis of covariance (ANCOVA), adjusting for differences in baseline outcome assessment scores. In analyzing the within-group and between-group intervention effects, both intent-to-treat (ITT) and per-protocol (PP) analyses were conducted, recognizing the strengths and limitations of each method. Within and between group effect sizes were computed using Cohen's *d* ($\text{Cohe}n^{\prime }s\;d=\frac{2\sqrt{\eta^{2}}}{\sqrt{1-\eta^{2}}}$; η^2^ is the partial eta square calculated from ANCOVA), with values of 0.2, 0.5, and 0.8 interpreted as small, medium, and large effect sizes, respectively (Cohen, 1988).

Clinically significant changes from baseline to post-intervention were examined using the Reliable Change Index (RCI), with an RCI score ≥ 1.95 considered indicative of clinically significant change (Jacobson and Truax, 1991). Using data from a study in Chinese BAI with 531 Chinese university students (i.e. Cronbach's *α* = 0.94 and *SD* = 16.04; Zhou et al., 2018), a change in BAI score (ΔBAI) of 9.94 from baseline to post-intervention was indicative of a clinically significant change in anxiety, whether improvement or worsening. Considering the cutoff point of BAI and RCI, four categories of clinically significant changes were identified: (1) Recovered: significant improvement in anxiety and minimal/no anxiety symptoms at post-intervention; (2) Improved: significant improvement in anxiety, but still experiencing mild or greater anxiety at post-intervention; (3) Deteriorated: significant decline in anxiety with mild or greater anxiety at post-intervention; (4) Unchanged: statistically insignificant changes in anxiety.

Variables related to the clinically significant improvement were identified using univariate logistic regression analysis, while variables related to the ΔFFMQ score were identified using ANOVA and Spearman's correlation analysis for categorical and continuous variables, respectively.

## 3 Research results

**Fig. 1:**
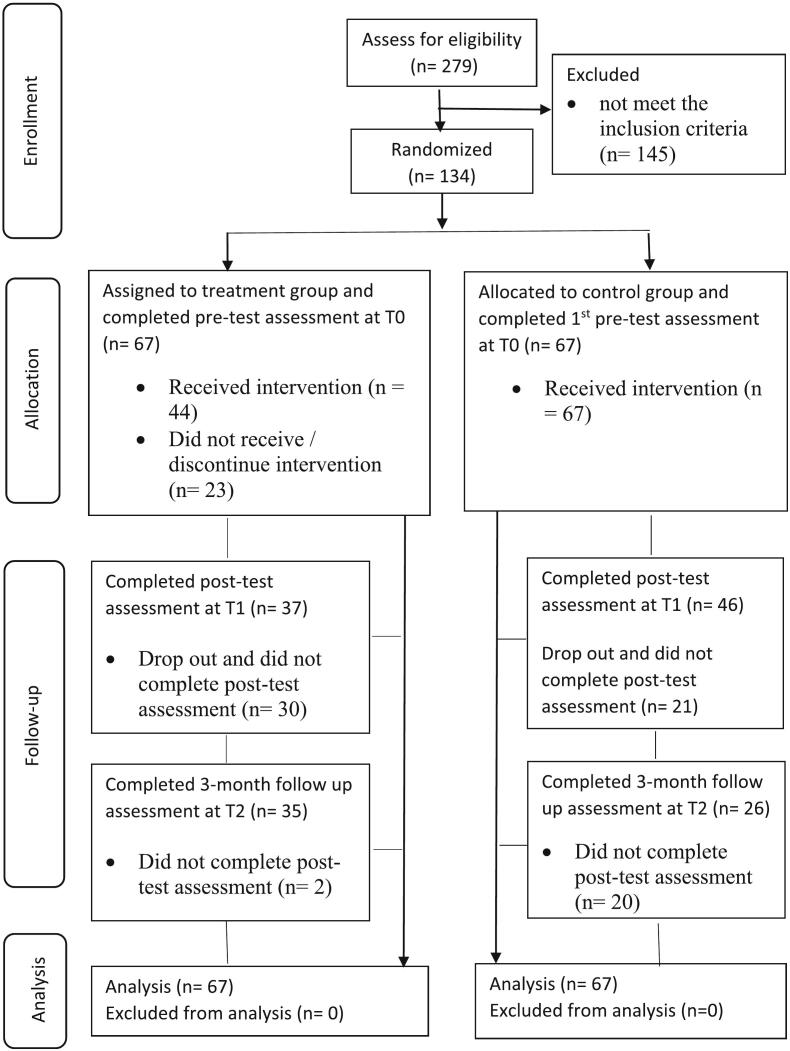
The CONSORT flow of participants through each stage of the study.

Please refer to Fig. 1 for participant progression through each stage of the study. The dropout rate for the intervention group was 44.78 % (*n* = 30) at post-intervention, and 47.67 % (*n* = 32) at the 3-month follow-up. *t*-Tests and chi-square tests revealed no significant differences between the dropout and retention groups for all baseline demographic variables and outcome measures, except gender. A higher percentage of females was observed in the retention group (90.36 %, *n* = 75) compared to the dropout group (74.51 %, *n* = 38). Missing data were assumed to be random and missing values were replaced by multiple imputation.

### 3.1 Participant characteristics

Independent *t*-tests and chi-square tests showed no significant differences in demographic variables between the intervention and control groups (Table 1). For all participants (*n* = 134), the mean age was 24.61 years (*SD* = 6.02). The majority were female (84.33 %, *n* = 113), single (89.55 %, *n* = 120), pursuing an undergraduate degree (68.66 %, *n* = 92), and living with their families (72.39 %, *n* = 97). Half relied on family financial support (51.49 %, *n* = 69). About one-eighth (12.69 %, *n* = 17) reported receiving a diagnosis of a mental disorder, primarily depression (5.97 %, *n* = 8), anxiety (5.22 %, *n* = 7), and bipolar disorder (1.49 %, *n* = 2).

**Table 1:** Baseline demographic and clinical characteristics of intervention and control group.

Characteristics	Intervention group	Control group	Total	*t*/χ^2^ (*p*)
	(*n* = 67)	*(n* = 67)	(*n* = 134)	
Age, mean ± *SD* (years)^a^	24.01 (4.93)	25.21 ± 6.92	24.61 (6.02)	−1.15 (0.25)
Female, *n* (%)^b^	57 (85.08)	56 (83.58)	113 (84.33)	0.06 (0.81)
Education level, *n* (%)^b^				2.22 (0.14)
Master degree	17 (25.37)	25 (37.31)	42 (31.34)	
Undergraduate	50 (74.63)	42 (62.69)	92 (68.66)	
Single, *n* (%)^b^	63 (94.03)	57 (85.08)	120 (89.55)	2.87 (0.09)
Main source of income, *n* (%)^b^				0.56 (0.91)
Family support	36 (53.73)	33 (49.25)	69 (51.49)	
Work	21 (31.34)	25 (37.31)	46 (34.33)	
Government financial support	7 (10.45)	6 (8.96)	13 (9.70)	
Saving	3 (4.48)	3 (4.48)	6 (4.48)	
Living situation, *n* (%)^b^				1.28 (0.53)
With family	50 (74.63)	47 (70.15)	97 (72.39)	
With co-tenants	12 (17.91)	11 (16.42)	23 (17.16)	
Alone	5 (7.46)	9 (13.43)	14 (10.45)	
Diagnosis (Nil), *n* (%)^b^	58 (86.57)	58 (86.57)	116 (86.57)	1.06 (0.59)
Clinical Anxiety (BAI ≥ 16), *n* (%)^b^	25 (37.31)	26(38.81)	51 (38.06)	0.03 (0.86)
Pre-BAI, mean ± *SD*^a^	15.24 ± 5.75	15.00 ± 6.65	15.12 ± 6.20	0.22 (0.82)
Pre-FFMQ, mean ± *SD*^a^	113.32 ± 13.17	116.12 ± 14.60	114.92 ± 13.94	−1.26 (0.21)

**Notes**: * = significant at *p* < 0.05; *p* *= p* value.

**Abbreviations**: χ^2^ = Pearson chi-square value; *t* = independent sample *t*-test value; BAI = Beck Anxiety Inventory; FFMQ = Five Facets Mindfulness Questionnaire.

a Independent sample *t*-test.

b Pearson Chi-Square.

### 3.2 Baseline outcome assessment scores

As shown in Table 1, independent *t*-tests revealed no significant difference in baseline BAI and FFMQ scores between the intervention and control groups. In general, participants reported a moderate level of anxiety with a mean BAI score of 15.12 (*SD* = 6.20). While over half (55.97 %, *n* = 75) exhibited mild anxiety (i.e., 7 < BAI score < 16), more than a third (38.06 %, *n* = 51) displayed clinical anxiety (i.e., BAI score ≥ 16). Additionally, participants reported a mean FFMQ score of 114.92 (*SD* = 13.94).

### 3.3 Adherence

Participant attendance rates were calculated based on the percentage of online modules completed and workshops attended, which totaled 16 h of mindfulness practice over an eight-week intervention period. Participation in a module and completion of the associated exercises and reflection questions were considered completion of a module. Among participants in the intervention group, the overall compliance rate was 49.92 %. Excluding those dropouts who did not receive any intervention shortly after being assigned to the intervention group (*n* = 23), about two-thirds completed all 16 online modules (63.64 %, *n* = 28) and at least one online mindfulness workshop via Zoom Apps (61.36 %, *n* = 27).

### 3.4 Primary intervention outcome – reduction in anxiety

#### 3.4.1 Pre- and post-intervention period

##### 3.4.1.1 Within group intervention effect

As shown in Table 2, the results of the paired-samples *t*-test in both the ITT and PP analyses indicated that the intervention group showed a significant reduction in the BAI score (ITT analysis: *t* = −5.58, *p* < 0.01; PP analysis: *t* = −5.99, *p* < 0.01), while the control group showed no significant reduction in the BAI score.

**Table 2:** Comparison of the changed outcome assessments scores within and between the intervention and control groups.

	Score T0 Mean ± *SD*	Score T1 Mean ± *SD*	Score T2 Mean ± *SD*	Within-group analysis		Between group analysis					
				T0-T1	T1-T2	T0-T1			T0-T2		
				t (*p*)	t (*p*)	F^a^	Partial η2	Cohen's d	F^b^	Partial η2	Cohen's d
Intent-to-treat analysis											
Intervention group											
BAI	15.24 ± 5.75	10.67 ± 5.28	9.74 ± 5.88	−5.58 (0.00)**	−1.27 (0.21)	7.08 (0.01)**	0.05	0.46	9.79 (0.00)**	0.07	0.55
FFMQ	113.32 ± 13.17	122.83 ± 12.62	121.97 ± 13.73	5.01 (0.00)**	−0.28 (0.79)						
Control group											
BAI	15.00 ± 6.65	13.42 ± 7.58	12.09 ± 5.91	−1.63 (0.11)	−1.37 (0.18)	8.09 (0.01)**	0.06	0.51	5.55 (0.02)*	0.04	0.41
FFMQ	116.43 ± 14.63	117.91 ± 12.60	120.50 ± 6.58	1.05 (0.30)	1.86 (0.07)						

Per-protocol analysis											
Intervention group											
BAI	15.24 ± 5.75	9.50 ± 6.15	9.26 ± 7.46	−5.99 (0.00)**	−0.20 (0.85)	8.74 (0.00)**	0.10	0.66	10.72 (0.00)**	0.16	0.87
FFMQ	113.32 ± 13.17	124.68 ± 16.40	123.34 ± 18.61	4.51 (0.00)**	−1.00 (0.33)						
Control group											
BAI	15.00 ± 6.65	13.78 ± 8.94	13.19 ± 8.64	−0.90 (0.37)	−0.50 (0.62)	8.31 (0.00)**	0.10	0.66	4.40 (0.04)*	0.07	0.55
FFMQ	116.43 ± 14.63	117.24 ± 15.38	118.88 ± 9.47	1.85 (0.70)	1.12 (0.27)						

**Notes**: * = significant at *p* < 0.05; ** = significant at *p* < 0.01.

**Abbreviations**: F^a^ = 2 (group) × 2 (time) repeated measures of ANCOVA adjusted for baseline outcome assessment; F^b^ = 2 (group) × 3 (time) repeated measures of ANCOVA adjusted for baseline outcome assessment; *SD* = standard deviation; ΔBAI = changed BAI score; ΔFFMQ = changed FFMQ score; BAI = Beck Anxiety Inventory; FFMQ = Five Facets Mindfulness Questionnaire. Partial η^2^ = Partial Eta Square; T0: pre-intervention; T1: post-intervention; T2: 3-month follow-up.

##### 3.4.1.2 Between group intervention effect

ANCOVA with 2 (group) × 2 (before vs. after) repeated measures in both the ITT and PP analyses indicated that the intervention group had a significantly greater reduction in BAI after adjusting for baseline BAI than the control group with a mean effect size (ITT analysis: *F* = 7.08, *p* < 0.01, Cohen's *d* = 0.46; PP analysis: *F* = 8.74, *p* < 0.01, Cohen's *d* = 0.66).

#### 3.4.2 3-Month follow-up

##### 3.4.2.1 Within group intervention effect

**Fig. 2:**
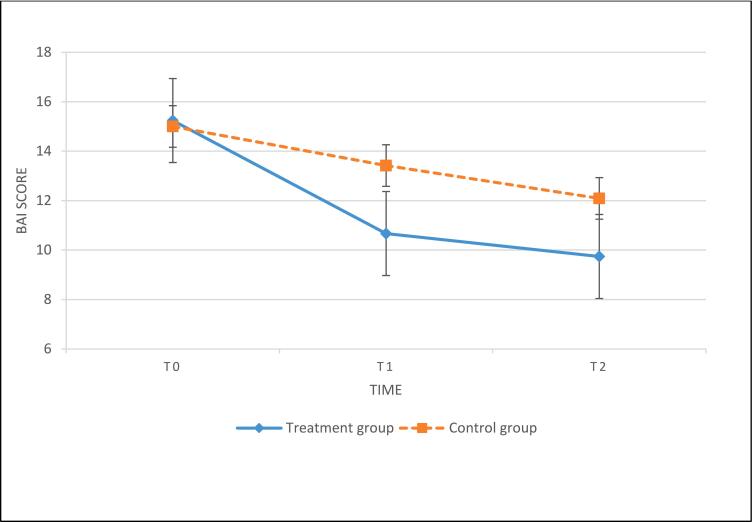
Change in BAI score from pre-intervention to 3-month follow-up with data based on the intent-to-treat analysis. **Abbreviations**: BAI = Beck Anxiety Inventory; T0: pre-intervention; T1: post-intervention; T2: 3-month follow-up.

**Fig. 3:**
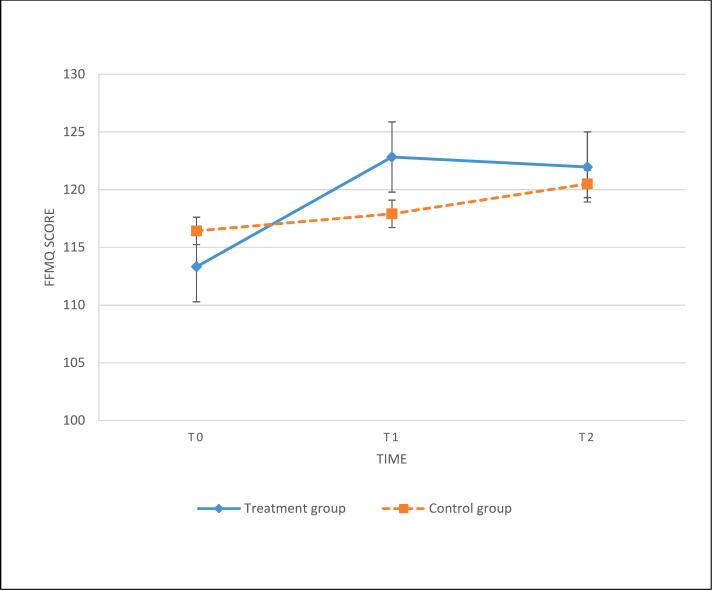
Change in FFMQ score from pre-intervention to 3-month follow-up with data based on the intent-to-treat analysis. **Abbreviations**: FFMQ = Five Facets Mindfulness Questionnaire; T0: pre-intervention; T1: post-intervention; T2: 3-month follow-up.

The results of the paired-samples *t*-test in the ITT and PP indicated that both the intervention and control groups had no significant change in the BAI score from T1 to T2. These results suggest that the improved BAI score at T1 was maintained at T2. Also see Fig. 2, Fig. 3.

##### 3.4.2.2 Between group intervention effect

ANCOVA with 2 (group) × 3 (time) repeated measures in both the ITT and PP analyses indicated that the intervention group had a significantly greater improvement in BAI score after adjusting for baseline BAI than the control group with a mean effect size (ITT analysis: *F* = 9.79, *p* < 0.01, Cohen's *d* = 0.55; PP analysis: *F* = 10.72, *p* < 0.01, Cohen's *d* = 0.87).

### 3.5 Secondary intervention outcome—improvement in mindfulness

#### 3.5.1 Pre- and post-intervention period

##### 3.5.1.1 Within group intervention effect

As shown in Table 2, the results of the paired *t*-test in both the ITT and PP analyses showed that the intervention group had a significant reduction in FFMQ score (ITT analysis: *t* = 5.01, *p* < 0.01; PP analysis: *t* = −4.51, *p* < 0.01), while the control group had no significant reduction in FFMQ score (Table 2).

##### 3.5.1.2 Between group intervention effect

The ANCOVA with 2 (group) × 2 (before vs. after) repeated measures in both the ITT and PP analyses showed that the intervention group had a significantly greater improvement in FFMQ score after adjusting for baseline FFMQ score than the control group with a mean effect size (ITT analysis: *F* = 8.09, *p* < 0.01, Cohen's *d* = 0.51; PP analysis: *F* = 8.31, *p* < 0.01, Cohen's *d* = 0.66).

#### 3.5.2 3-Month follow-up

##### 3.5.2.1 Within group intervention effect

The results of the paired-samples t-test in the ITT and PP indicated that both the intervention and control groups had no significant change in FFMQ score from T1 to T2. These results indicated that the improved FFMQ score after the intervention was maintained at T2. Also see Fig. 2, Fig. 3.

##### 3.5.2.2 Between group intervention effect

ANCOVA with 2 (group) × 3 (time) repeated measures in both the ITT and PP analyses indicated that the intervention group had a significantly greater improvement in FFMQ score after adjustment than the control group with a small to medium effect size (ITT analysis: *F* = 5.55, *p* < 0.05, Cohen's *d* = 0.41; per-protocol analysis: *F* = 4.40, *p* < 0.05, Cohen's *d* = 0.55).

### 3.6 Clinically significant change

After completing the intervention, one-third (34.33 %, *n* = 23) had achieved clinically significant improvement (i.e., BAI > 9.94) at post-intervention. Specifically, 11.94 % (*n* = 8) of the intervention group were regarded as recovered, 22.39 % (*n* = 15) improved, 61.19 % (*n* = 41) had no significant change, and 4.48 % (*n* = 3) deteriorated.

### 3.7 Characteristics of participants who benefited most from low-intensity iMBI

As shown in Table 3, the results of univariate logistic regression showed that the clinically significant improvement was not correlated with all demographic variables, but was correlated with baseline BAI score (*W* = 1.73, *p* < 0.01), having clinical anxiety at baseline (*W* = 10.69, *p* < 0.01), and baseline FFMQ score (*W* = 6.15, *p* < 0.05).

**Table 3:** Correlation between baseline demographic and clinical variables with the intervention outcomes for the intervention group (n = 67).

Variables	Clinically significant improvement	Δ FFMQ	
	Wald (*p*)	F (p)	ρ (p)
Gender	1.03 (0.31)	2.49 (0.12)	
Marital status	−0.16 (0.69)	0.20 (0.65)	
Education level	0.01 (0.92)	0.65 (0.06)	
Source of income	3.31 (0.51)	5.67 (0.01)**	
Living situation	0.59 (0.75)	1.66 (0.20)	
Diagnosis	1.51 (0.83)	0.69 (0.60)	
Clinical anxiety (BAI ≥ 16)	10.69 (0.00)**	0.93 (0.34)	
Age	0.37 (0.54)		0.26 (0.03)*
Baseline BAI	1.73 (0.00)**		0.20 (0.10)
Baseline FFMQ	6.15 (0.01)*		−0.63 (0.00)**

**Notes**: * = significant at *p* < 0.05; ** = significant at *p* < 0.01.

**Abbreviations**: ρ = Spearman's rho; ΔBAI = changed BAI score from pre-intervention (T0) to post-intervention (T1); ΔFFMQ = changed FFMQ score from pre-intervention (T0) to post-intervention (T1); BAI = Beck Anxiety Inventory; Clinically significant improvement = A reduction in BAI score ≥ 9.94; FFMQ = Five Facets Mindfulness Questionnaire.

Additionally, the ANOVA and Spearman's correlation analysis showed that the ΔFFMQ score was not correlated with all, except one, demographic variables. Those participants having a full-time or part-time job and relying on their salary to support their living showed a more significant reduction in FFMQ score (*M* = −9.07, *SD* = 9.13) than those relying on family financial support (*M* = −3.73, *SD* = 7.07) and those relying on governmental financial support (*M* = −1.26, *SD* = 6.37). Also, the ΔFFMQ score was correlated positively with age (*r* = 0.26, *p* < 0.05), and negatively with baseline FFMQ total score (*r* = −0.63, *p* < 0.01).

## 4 Discussion

The demographic characteristics of participants in this study closely align with those of previous surveys on the prevalence of anxiety among university students in Hong Kong during the COVID-19 pandemic (Hung et al., 2022; Shek et al., 2022), except that our study included a higher percentage of participants pursuing a master's degree.

This randomized controlled trial examined the effectiveness of a low-intensity iMBI for anxious university students. The program comprised sixteen self-paced online modules, accessible anywhere, anytime and anonymously via an online platform, and two half-day online mindfulness workshops delivered via Zoom apps over an eight-week period. The results of our study confirm the feasibility and effectiveness of a low-intensity iMBI to reduce anxiety symptoms and promote mindfulness, with a medium effect size at post intervention. Furthermore, the effects of the intervention in reducing anxiety and promoting mindfulness were maintained at the three-month follow up. Furthermore, this study is one of the few studies to show that low-intensity iMBI produces clinically significant improvement in participants with anxiety.

Specifically, this low-intensity iMBI achieved a medium effect size in reducing anxiety (Cohen's *d* = 0.46), compared to previous research on iMBIs during the pre-COVID-19 pandemic (Cohen's *d* = 0.26; Sommers-Spijkerman et al., 2021) and during the pandemic (Cohen's *d* ranging from 0.31 (Karing, 2022) to 0.72 (Sun et al., 2022)). In addition, this low-intensity iMBI yielded a medium effect size in enhancing mindfulness (Cohen's *d* = 0.51), compared to previous studies on iMBIs during the pre-COVID-19 pandemic (Cohen's *d* ∼ 0.40; Sommers-Spijkerman et al., 2021) and during the pandemic (Cohen's *d* ranging from 0.36 (Sun et al., 2022) to 2.09 (Karing, 2022)).

Several characteristics of this low-intensity iMBI may have contributed to its increased effectiveness in reducing anxiety and improving mindfulness. These include availability to participants who suffered emotional distress during the COVID-19 pandemic in Hong Kong, targeting Asian university students who are highly computer literate (Schmidt et al., 2019), guidance through online mindfulness workshops (Sommers-Spijkerman et al., 2021), and a comprehensive intervention duration of eight weeks (Witarto et al., 2022).

To our knowledge, very few studies on iMBIs report their effects on facilitating clinically significant improvement. This study demonstrated that the low-intensity iMBI promoted clinically significant improvement in participants with anxiety. Specifically, one-tenth of participants were regarded as recovered, about one-fourth showed improvement, and only a small fraction (<5 %) deteriorated. The small deterioration rate observed in our study is consistent with previous research on Internet interventions (Rozental et al., 2017). However, further research is needed to validate these findings.

In this study, participants with clinical anxiety benefited more, exhibiting greater reductions in anxiety, a finding supported by previous research (Sommers-Spijkerman et al., 2021; Witarto et al., 2022). Furthermore, older participants, those relying on their work salary for living expenses, and those with lower levels of mindfulness at baseline showed more improvement in mindfulness. Given that there are limited studies identifying those who benefit most from iMBIs, additional research in this area is warranted.

For professionals seeking to promote iMBI programmes for university students internationally, several aspects of this low-intensity iMBI are worth noting. First, this low-intensity iMBI overcame many barriers associated with face-to-face MBI by providing an accessible, evidence-based online counselling platform that is available anywhere, anytime and anonymously (Witarto et al., 2022). During the COVID-19 pandemic, university students showed higher engagement with iMBI compared to in-person support groups (Sun et al., 2022). The short, structured self-study modules with concise homework assignments were found to best meet the needs of university students who lead busy lives (Young et al., 2022b). In addition, the low-intensity iMBI allowed participants to learn the online modules and attend the online workshops anonymously, eliminating the need for face-to-face meetings with counsellors, which reduced potential stigma (Young et al., 2020; Young et al., 2022a).

Second, by adapting from Mindfulness-based Cognitive Therapy (Segal et al., 2013), this iMBI could address the needs of university students during the COVID-19. Anxiety during the pandemic was triggered by various factors such as fear of infection, quarantine, precautions, COVID-19 information overload, online learning, financial hardship, living alone, and disruption of daily routines (Choi et al., 2020; Hung et al., 2022; Liyanage et al., 2022; Shek et al., 2022). By adapting from Mindfulness-based Cognitive Therapy (Segal et al., 2013), this guided iMBI aimed to raise awareness of habitual negative thinking patterns, promote presence in the present moment rather than rumination, support acceptance of emotional distress, and promote a sense of hope in the face of stressful situations (Segal et al., 2013). This enabled participants to face stress, unexpected life changes and the challenges of the COVID-19 pandemic.

Third, non-adherence has been a major problem with iMBI, as it could limit efficacy. Rates of non-adherence of up to 65 % have been reported in iMBI trials (Sommers-Spijkerman et al., 2021). In our study, a non-adherence rate of 50 % was observed in the intervention group. This is comparable to previous iMBI studies prior to COVID-19, which reported adherence rates of 18 % to 65 % (Sommers-Spijkerman et al., 2021), but higher than the ≤14 % reported during the COVID-19 pandemic (Witarto et al., 2022). Strategies to promote adherence to these low-intensity iMBIs could include automated and staff support, such as offering weekly video conferencing (Karing, 2022) and regular group practice (Cheng et al., 2022). Given recent advances in artificial intelligence (AI), future implementations of iMBI could explore the possibility of integrating AI to improve adherence and engagement, possibly through personalized conversational agents or AI-driven interventions, as suggested by Carlbring et al. (2023).

The current study has several methodological limitations. First, our sample was recruited from a single university and thus may not be representative of the entire higher education population in Hong Kong. Second, the high dropout rate may lead to biased estimates of intervention effects. However, the dropout and retention groups were quite similar, and using a multiple imputation method to deal with missing data may mitigate this bias. Third, the inclusion of the inactive control group as a comparison group may lead to biased estimates of intervention effects. Future large-scale randomized controlled trials with an active control group and longer-term follow-up in populations with different demographic and clinical characteristics are needed to validate the effectiveness of these iMBIs in different cultures and societies.

## CRediT authorship contribution statement

YOUNG Kim-wan: Study concept and design, negotiating with other social service organizations involved in this project, acquisition of subjects and data, analysis and interpretation of data, and preparation of manuscript.

Per CARLBRING: Interpretation of data, Preparation and review of manuscript.

Petrus NG: Negotiating with other social service organizations involved in this project, and preparation of manuscript.

CHENG Yi Ting: Preparation and review of manuscript.

CHEN Qi-rong: Collection of data, Review of manuscript.

Ng Siu-man: Review of manuscript.

## Funding support

This research study is funded by 10.13039/501100001747Hong Kong Baptist University (Reference no.: RC-FNRA-IG/19-20/SOSC/06).

## Declaration of competing interest

The authors have no conflict of interest to report.
